# Resource-Efficient Fusion with Pre-Compensated Transmissions for Cooperative Spectrum Sensing

**DOI:** 10.3390/s150510891

**Published:** 2015-05-08

**Authors:** Dayan Adionel Guimarães, Guilherme Pedro Aquino, Marco E. G. V. Cattaneo

**Affiliations:** 1 National Institute of Telecommunications—Inatel, Av. João de Camargo, 510, 37540-000 Santa Rita do Sapucaí, Brazil; E-Mail: dayan@inatel.br; 2 University of Hull, Cottingham Road, Hull HU6 7RX, UK; E-Mail: m.cattaneo@hull.ac.uk

**Keywords:** cognitive radio, cooperative spectrum sensing, decision fusion, channel inversion, crest factor, clipping

## Abstract

Recently, a novel fusion scheme for cooperative spectrum sensing was proposed for saving resources in the control channel. Secondary users (SUs) simultaneously report their decisions using binary modulations with the same carrier frequencies. The transmitted symbols add incoherently at the fusion centre (FC), leading to a larger set of symbols in which a subset is associated with the presence of the primary user (PU) signal, and another subset is associated with the absence of such a signal. The decision criterion applied for discriminating these subsets works under the assumption that the channel gains are known at the FC. In this paper, we propose a new simultaneous transmission and decision scheme in which the task of channel estimation is shifted from the FC to the SUs, without the need for feeding-back of the estimates to the FC. The estimates are used at the SUs to pre-compensate for the reporting channel phase rotations and to partially compensate for the channel gains. This partial compensation is the result of signal clipping for peak-to-average power ratio (PAPR) control. We show, analytically and with simulations, that this new scheme can produce large performance improvements, yet reduces the implementation complexity when compared with the original one.

## 1. Introduction

The problem of the scarcity of free bands in the radio frequency spectrum is known to be a severe obstacle to the deployment of existing wireless communication systems, as well as to the development of new ones. A promising solution for alleviating such a problem has come with the advent of cognitive radio (CR) [1] technology, which is a candidate for being one of the key enabling alternatives in the fifth-generation (5G) wireless communication systems [2]. In one of the CR concepts, unused spectrum portions allocated to the primary (incumbent) network can be opportunistically used by secondary CR networks. In order to accomplish this task, a spectrum sensing [3] technique detects the unused frequency bands before opportunistic access by the CRs. As soon as the incumbent re-establishes the occupation of that band, the secondary network must be capable of detecting the event and vacate the band before harmful interference is caused to the primary network.

In order to increase the reliability of the decisions regarding the occupancy of a given channel, cooperative spectrum sensing [3] has become a common choice. In decision fusion cooperative spectrum sensing, individual CR or secondary user (SU) decisions are sent to a fusion centre (FC), where the final decision upon the channel state is made. A well-known decision fusion is the *K*-out-of-*M* rule, in which the FC decides the presence of a primary user (PU) when at least *K* among *M* secondary users declare an active PU in the band of interest. To send their local decisions to the FC, the SUs make use of a reporting control channel, typically adopting a time division multiple access (TDMA), a frequency division multiple access (FDMA) or a combination of these. However, as the number of SUs grows, these multiple access techniques tend to require more time or frequency resources, reducing the overall spectral efficiency of the decision fusion task.

A number of attempts have been made for saving bandwidth during the reporting of secondary user's decisions. For instance, in [4], the authors propose a method for decreasing the average number of sensing bits sent to the FC. In this method, only the users with high reliability are allowed to report their binary decisions. A cooperative sensing without a dedicated reporting channel is proposed in [5]. In this proposal, the SUs send their local decisions to the FC in the same licensed primary user channel, potentially causing interference to the primary network; the authors of [5] suggest in [6] how to mitigate this interference. In [7], each SU reports its local decision only after having enough confidence. As a consequence, the SUs report their decisions in a different time, decreasing the necessary maximum bandwidth in the reporting channel. In [8,9], the FC coordinates the reporting task in the SU network, choosing the SUs that will report their decisions. These SUs then randomly send their decisions until the condition required to make a global decision is satisfied at the FC. As a result, the number of sensing reports is reduced. In [10], each SU shares the local decision with its neighbour, sequentially and in a ring basis, and the last SU makes the final decision about the PU signal presence. In [11], the authors propose a final decision weighting scheme with a low average number of sensing bits sent through the reporting channel.

The energy efficiency is also an important aspect of the fusion task of the sensors' data, since it is also desired to save the energy used for signal processing and transmission. Initiatives that take into account the energy efficiency are presented, for example, in [12–19].

In the recent technique proposed in [20], *M* secondary users send their local decisions to the FC simultaneously and at the same frequency, improving the resource efficiency of the reporting channel transmissions. The transmitted decisions add incoherently at the FC, forming a set of 2*^M^* symbols (if fading reporting channels are considered) or *M* + 1 symbols (in the case of non-fading channels), in which a subset is associated with the presence of the PU signal and another subset is associated with the absence of the PU signal. The decision criterion applied for discriminating these subsets works under the assumption that the channel gains from the SUs to the FC are known at the FC. The author of [20] suggests that the estimation of these gains can be accomplished in practice by means of sounding techniques in the uplink. Extensions and further details about the technique proposed in [20] are provided in [21]. A novel decision rule is proposed in [22] in order to improve the performance of the technique suggested in [20], with practically no additional cost in complexity.

Following [20], in [21,22], it is assumed that the channel gain estimates used in the global decision process are obtained from some pilot signal sent in the downlink. Moreover, the transmitted and received signal model adopted in [21,22] follows the one suggested in [20]. In this paper, we propose a new simultaneous transmission scheme in which the task of channel estimation is shifted from the uplink to the downlink, eliminating the use of resources for feeding back the estimates to the FC. This task, besides being more suitable to the purpose of resource savings than the one suggested in [20], can be easily accomplished in practice, as long as the coherence time of the channel is larger than the time lag between the downlink sounding signal transmission and the uplink reporting transmissions. In our new fusion scheme, the channel estimates are used by each SU to pre-compensate for the channel phase rotation and to partially compensate for the reporting channel gain. This partial compensation results from signal clipping, which is suggested here as a means for peak-to-average power ratio (PAPR) control or, equivalently, crest factor (CF) control: without this control, high PAPR or CF levels would result from the compensation of low channel gains. We show that our new scheme can achieve large performance improvements over a fading reporting channel, yet reducing the system implementation complexity when compared with the original one. The performance and complexity gains come as a consequence of the simplification of the received constellation and the corresponding decision rule, when compared to [20], to a situation very close to the one that is achieved when the reporting channels are in a non-fading condition. We also give expressions for computing the key performance metrics of our technique. Such expressions, though derived through approximations, can produce accurate results, which are validated with simulations.

The remainder of the paper is organized as follows: The system model is presented in Section 2, where the proposed transmission technique and its decision rule are also described, and the performance metrics are derived. Theoretical and numerical results are provided and discussed in Section 3. Finally, Section 4 concludes the paper.

## 2. System Model

We consider a cooperative spectrum sensing system with *M* secondary users that transmit their local hard decisions to the fusion centre using binary phase-shift keying (BPSK) modulation, at the same time and carrier frequency. Let *m_k_* represent the binary local decision generated by the *k*-th secondary user, with *m_k_* = 1 indicating the presence of a primary user signal (hypothesis *H*_1_) and *m_k_* = 0 indicating no active primary user (hypothesis *H*_0_). In the subsequent subsections, we first present the original transmission and decision rules proposed in [20]. Then, we describe the new transmission and decision rules proposed in this paper.

### 2.1. Original Fusion Scheme

In [20], the baseband equivalent of the BPSK symbols with energy *E*_b_ transmitted from each SU are $s_{k}=\left(2m_{k}-1\right)\sqrt{E_{\text{b}}}$. If *h_k_* is the complex gain of the reporting channel between the *k*-th secondary user and the FC, the received signal sample at the FC is given by:

(1) $$r=\overset{M}{\underset{k=1}{\text{∑}}}h_{k}s_{k}+n$$

where *n* is the zero-mean, additive white Gaussian noise (AWGN) sample with variance *σ^2^* and power spectral density *N*_0_ = 2*σ*^2^ watts/hertz.

It is assumed in [20] that the complex channel gains are known at the FC, which allows the final decision upon the sensed channel state to be reached from *r*. Specifically, define a local decision vector **s** = [*s*_1_, *s*_2_,…, *s_m_*]^T^ and a channel vector **h** = [*h_1_,h_2_*,…,*h_m_*]^T^, with [·]^T^ meaning transposition. Let *D*_0_ and *D*_1_ represent the sets of local decision vectors that would lead to the choice of *H*_0_ and *H*_1_, respectively, on the basis of the *K*-out-of-*M* rule. According to the decision rule proposed in [20], the FC will choose *H*_1_ if:

(2) $$\underset{\text{s}\in D_{1}}{\text{∑}}\exp\left\{-\frac{{\left|r-{\text{h}}^{\text{T}}\text{s}\right|}^{2}}{2\sigma^{2}}\right\}\geq \underset{\text{s}\in D_{0}}{\text{∑}}\exp\left\{-\frac{{\left|r-{\text{h}}^{\text{T}}\text{s}\right|}^{2}}{2\sigma^{2}}\right\}$$

and will choose *H*_0_ otherwise. This can be classified as a maximum *a posteriori* (MAP) decision rule with uniform *a priori* probabilities for the local decision vectors, although it is denoted as a maximum-likelihood (ML) decision rule in [20].

Expressions for computing the global probabilities of detection, *P*_D,FC_, and false alarm, *P*_FA,FC_, at the FC are provided in [20] for the simplest case of *h_k_* = 1. However, such expressions are accurate only in the high SNR regime [21], since they were derived considering an approximated version of Equation (2), which applies the analytic approximation of the max function to the log-sum-exp (logarithm of the sum of exponentials) function [23], *i.e.*, $\ln{\text{∑}}_{i}\exp\left(a_{i}\right)≃\max\left(a_{i}\right)$.

One of the strongest reasons for the difficulty in deriving expressions for *P*_D,FC_ and *P*_FA,FC_ when the reporting channel gains are complex and time-varying is the fact that the received symbol constellation at the FC shows-up with randomly varying shapes and symbol positions, leading to decision regions that vary accordingly [21]. The derivation of *P*_D,FC_ and *P*_FA,FC_ in this case remains an open problem.

### 2.2. Proposed Fusion Scheme

As in [20], we assume that the complex channel gains from the SUs to the FC are known. However, unlike in [20], these channel gains are known only at the SUs, not at the FC. In practice, the estimation of these gains can be made by means of a sounding signal periodically broadcasted by the FC (in the downlink). Accurate estimates of the uplink complex gains can be made as long as the coherence time of the channel is larger than the time lag between the downlink sounding signal transmissions and the uplink reporting transmissions, and the sounding signal carrier frequency is equal or approximately equal to the carrier frequency used for the reporting transmissions. For a sufficiently slow fading channel, the period of the sounding signal transmissions can be large, leading to a low average transmission rate. Recall that, in the case of [20], to use the idea of estimating the reporting channel gains at the FC by means of sounding signals in the uplink would require a larger amount of resources, since the probing signal sent from each SU would have to be orthogonal to the remaining ones.

The channel estimates in our approach are used by each SU to pre-compensate for the reporting channel phase rotation and to partially compensate for the corresponding channel gains in a channel-inversion fashion, as described in the sequel. As far as we know, no similar idea has been applied yet to the fusion scheme proposed in [20]. As will be shown later on in this paper, this pre-compensation leads to a very simple decision rule at the FC and to a performance that beats the one achieved by the original scheme of [20]. These are the main specific advantages of our proposal.

Let the complex reporting channel gains be written as *h_k_* = *α_k_e^jθ_k_^*. Let us define the clipping threshold $C=c\sqrt{E_{\text{b}}}$, where *c* is a positive constant. This threshold will act by limiting the energy of the transmitted symbols at low values of the estimated channel gains {*α_k_*}. Such a process is necessary in practice as a means for PAPR control, since, without this control, high PAPR levels that would result from the compensation of low channel gains would impose a strong limitation to the design of the power amplifiers, which would have to work under low power efficiency and very wide, perhaps prohibitive, dynamic ranges.

By the influence of channel compensation and clipping, a symbol transmitted by the *k*-th SU can be written as:

(3) $$s_{k}=\left(2m_{k}-1\right)\min\left(\frac{1}{\alpha_{k}},C\right)e^{-j\theta_{k}}\sqrt{\frac{E_{\text{b}}}{\xi }}$$

where the factor $\sqrt{E_{\text{b}}/\xi }$ ensures that the average energy per symbol is maintained in *E*_b_. The variable *ξ* is the second moment of the random variable *y_k_* = min(1/*α_k_*, *C*). If {*α_k_*} are Rayleigh-distributed, *ξ* can be computed from:

(4) $$\xi =\int_{0}^{\infty }\min^{2}\left(\frac{1}{2},C\right)\frac{2z}{\Omega }\exp\left(-\frac{z^{2}}{\Omega }\right)dz$$

where Ω is the second moment of the Rayleigh fading magnitude. By noticing that:

(5) $$\min\left(1/z,C\right)=\{\begin{array}{cc} 1/z, & 0\leq 1/z\leq C\\ C, & C\leq 1/z\leq \infty \end{array}$$

Equation (4) can be written as:

(6) $$\begin{array}{cc} \xi & =\int_{1/C}^{\infty }\frac{2}{z\Omega }\exp\left(-\frac{z^{2}}{\Omega }\right)dz+\int_{0}^{1/C}C^{2}\frac{2z}{\Omega }\exp\left(-\frac{z^{2}}{\Omega }\right)dz\\ & =E_{1}\;\left(\frac{1}{C^{2}}\right)+C^{2}\;\left[1-\exp\left(-\frac{1}{C^{2}\Omega }\right)\right] \end{array}$$

where E_1_(·) is the generalized exponential integral function of order one.

The transmitted symbols defined in Equation (3) define a rotated and scaled BPSK constellation that can be easily generated in practice by means of a quadrature transmitter [24].

Substituting *h_k_* = *α_k_e^jθ_k_^* and *s_k_* from Equation (3) in Equation (1), the received signal sample at the FC now becomes:

(7) $$r=\overset{M}{\underset{k=1}{\text{∑}}}\left(2m_{k}-1\right)\alpha_{k}\min\left(\frac{1}{\alpha_{k}},C\right)\sqrt{\frac{E_{\text{b}}}{\xi }}+n$$

Since we have made a phase pre-compensation at the SUs, the received signals at the FC add coherently and, as a consequence, the received signal samples are real-valued. These samples can be obtained from the in-phase branch only (real part) in a quadrature receiver. The quadrature branch need not be used.

If the clipping threshold *C* → ∞, meaning no clipping, from Equation (7), it can be seen that the noiseless received symbols will be a sum of *M* Bernoulli random variables, thus having a binomial distribution with *M* + 1 real-valued symbols. The *i*-th symbol level is given by $\left(2i-M-2\right)\sqrt{E_{\text{b}}/\xi }$, *i* = 1, …, *M* + 1. The *K* symbols with the smallest values correspond to the choice of *H*_0_ and the remaining *M* + 1 − *K* symbols to the choice of *H*_1_ according to the *K*-out-of-*M* rule. The probability of the *i*-th symbol is given by:

(8) $$P_{i}=\left(\begin{array}{c} M\\ i-1 \end{array}\right)\;p^{i-1}{\left(1-p\right)}^{M-i+1}$$

with *p* being the probability of success of the Bernoulli random variables, *i.e.*, *p* = *P*_D,SU_ or *p* = *P*_FA,SU_, where *P*_D,SU_ and *P*_FA,SU_ are, respectively, the probability of detection and the probability of false alarm at each secondary user terminal.

**Figure 1:**
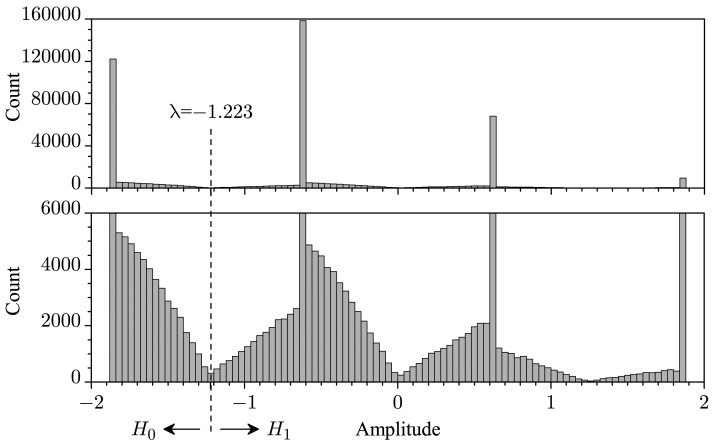
Histogram of the noiseless received symbols for *M* = 3, *E*_b_ = 1, Ω = 1, $C=3\sqrt{E_{\text{b}}}$ and *p* = 0.3. The value of λ is for *K* = 1. The lower part is a rescaled version of the upper one.

As the value of *C* is reduced, the effect of clipping in the symbols transmitted by the SUs starts to be noticed in *r*. As an example, Figure 1 shows, in its upper part, the histogram of the noiseless received symbols for *M* = 3, *E*_b_ = 1, Ω = 1, $C=3\sqrt{E_{\text{b}}}$ and *p* = 0.3, for 500,000 realizations of the channel gains and of the vectors with the SU's decisions. The lower part of the figure is just a rescaled version of the upper part. From this figure, one can notice that symbols having values other than the awaited ones $\left(2i-M-2\right)\sqrt{E_{\text{b}}/\xi }$ are appearing, with a probability that depends on the clipping threshold: the larger the threshold, the lower the probability. The probability that the noiseless received symbol values fall outside the awaited ones is given by the probability of clipping in one or more SUs, that is,

(9) $$P_{\text{out}}=1-{\left(1-P_{\text{clipp}}\right)}^{M}$$

where *P*_clipp_ is the probability of the occurrence of clipping in a single SU. Considering a Rayleigh fading channel, this probability can be computed from:

(10) $$P_{\text{clipp}}=\int_{0}^{1/C}\frac{2z}{\Omega }\exp\;\left(-\frac{z^{2}}{\Omega }\right)dz=1-e^{-\frac{1}{C^{2}\Omega }}$$

From Figure 1, one can also notice a very important aspect of our proposal. Observe that the decision at the FC can be made based on a simple comparison of the received sample with a threshold value that is exactly computed from:

(11) $$\lambda =\left(2K-M-1\right)\sqrt{\frac{E_{\text{b}}}{\xi }}$$

As an example, again considering Figure 1, if *K* = 1, we will have λ = −1.223, the midpoint between the leftmost noiseless symbol value, which refers to *H*_0_, and its neighbour, which refers to *H*_1_ along with the remaining ones located on its right side.

A natural question now arises: which is the optimum value of the clipping threshold? To answer this question, we must first notice that, from the perspective of the spectrum sensing performance, the value of *C* would have to be as large as possible so that *P*_out_ becomes small. However, large values for *C* would result in a prohibitively high PAPR for the signals transmitted by the SUs, since transmitted symbols with high energy would appear whenever low estimated channel gains were used in the pre-compensation process. Clearly, we have a trade-off problem in the choice for *C*. To solve this problem, let us first define a PAPR measure, which from Equation (3) can be written as:

(12) $$\text{PAPR}=\frac{{\left\|\text{s}\right\|}_{\infty }^{2}}{\text{E}\left[{\left\|\text{s}\right\|}_{2}^{2}\right]}=\frac{{\left(C\sqrt{E_{\text{b}}/\xi }\right)}^{2}}{E_{\text{b}}}=\frac{C^{2}}{\xi }$$

where s = [*s*_1_, *s*_2_,…, *s_M_*]^T^, with [·]^T^ meaning transposition, ‖s‖_∞_ is the Chebyshev norm of s, ‖s‖_2_ is the Euclidean norm of s and 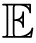
[·] denotes the expected value operation.

**Figure 2:**
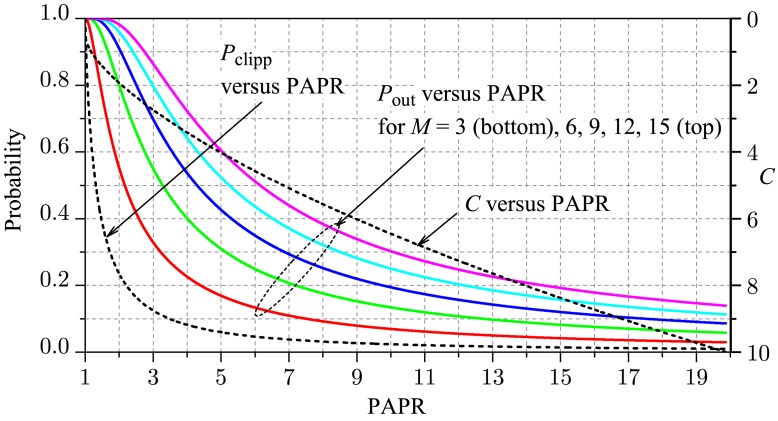
*P*_clipp_ and *P*_out_ *versus* PAPR for 0 < *C* < 10, *M* = 3, *E*_b_ = 1 and Ω = 1.

We then plot Equations (9) and (10) against Equation (12) for several values of *C*, as illustrated in Figure 2, for *M* = 3, *E*_b_ = 1 and Ω = 1. Being trade-off curves, any point over each of them is as good as any other point. However, notice that *P*_clipp_ reduces slowly as the PAPR increases, for *P*_clipp_ ≤ 0.1. If *P*_clipp_ = 0.1, then PAPR ≈ 3.5 and *P*_out_ ≈ 0.3 for *M* = 3. Then, adopting the reference value of *P*_out_ = 0.3 for determining the clipping threshold will guarantee *P*_clipp_ < 0.1 for *M* > 3. Hereafter, the clipping threshold *C* will be computed by inverting Equations (9) and (10) for *P*_out_ = 0.3. It will be shown that this approach leads to an attractive trade-off solution between the spectrum sensing performance and the PAPR of the signals transmitted by the SUs.

It is informative to mention that a very similar approach was used to determine the time-bandwidth product of the Gaussian filter adopted for the GMSK (Gaussian-filtered minimum shift keying) modulation in the GSM (global system for mobile) standard. In this case, by the influence of the time-bandwidth product, spectral compactness was traded against the performance degradation caused by the inter-symbol interference introduced by the filter ([24], Section 6.6.3).

It is also informative to mention that a BPSK signal filtered with a root-raised-cosine filter can easily reach PAPR levels above 3.5, up to 10 or more, for low roll-off factors [25]. Since the transmission of any modulated signal is almost always accomplished after filtering, for spectrum control purposes, it is immediate to conclude that PAPR values even above 3.5 can be produced in the original fusion scheme proposed in [20], just due to the filtering effect on the transmitted BPSK signal.

### 2.3. Probabilities of Detection and False Alarm

The received symbols at the FC are corrupted by Gaussian noise, but their actual positions in the received constellation are also dependent on the clipping threshold and channel fading, as well as on the decisions made by the SUs. Under the assumption of constant channel gains during the reporting phase, the Gaussian noise is the single short-term influence on the received symbols. Then, the global probabilities of detection and false alarm, respectively *P*_D,FC_ and *P*_FA,FC_, under clipping, fading and noise can be computed by averaging the corresponding probabilities computed under the noise-only condition, over the probability density function (pdf) related to the joint effect of the fading and clipping. Thus, the derivation of *P*_D,FC_ and *P*_FA,FC_ starts with the derivation of the pdf of the noiseless part of Equation (7).

A symbol transmitted by the *k*-th SU, at the fusion centre and without noise, can be written as:

(13) $$\rho_{k}=\left(2m_{k}-1\right)\min\left(\frac{1}{\alpha_{k}},C\right)\alpha_{k}\sqrt{\frac{E_{\text{b}}}{\xi }}$$

Since clipping occurs when *1*/*α_k_* > *C*, *ρ_k_* will be a scaled two-sided, truncated $\left(\text{at}\pm \sqrt{E_{\text{b}}/\xi }\right)$ Rayleigh random variable with values $\pm C\alpha_{k}\sqrt{E_{\text{b}}/\xi }$ occurring with probability *P*_clipp_, combined with a uniform random variable with values $\pm \sqrt{E_{\text{b}}/\xi }$ and probability 1 − *P*_clipp_. Let us define:

(14) $$L=\sqrt{E_{\text{b}}/\xi }$$

and define the scaled Rayleigh pdf:

(15) $$g\left(z\right)=\{\begin{array}{cc} \frac{2z}{\Omega C^{2}L^{2}}\exp\left(-\frac{z^{2}}{\Omega C^{2}L^{2}}\right) & \text{if}z\geq 0\\ 0 & \text{if}z<0 \end{array}$$

and an area scaling parameter:

(16) $$A=\int_{0}^{L}g\left(z\right)dz$$

Notice that if *L* is made equal to 1/*C* in Equation (16), it turns out that this equation becomes equal to Equation (10), meaning that *A* = *P*_clipp_. This equality is used in the derivations hereafter.

From above, the pdf of *ρ_k_* can be written as,

(17) $$\begin{array}{cc} f_{\rho k}\left(\rho_{k}\right) & =\frac{P_{\text{clipp}}}{A}\left[1-u\left(\left|\rho_{k}\right|-L\right)\right]\;\left[pg\left(\rho_{k}\right)+\left(1-p\right)g\left(-\rho_{k}\right)\right]+\left(1-P_{\text{clipp}}\right)\left[p\delta \left(\rho_{k}-L\right)+\left(1-p\right)\delta \left(\rho_{k}+L\right)\right]\\ & =\left[1-u\left(\left|\rho_{k}\right|-L\right)\right]\;\left[pg\left(\rho_{k}\right)+\left(1-p\right)g\left(-\rho_{k}\right)\right]+\left(1-P_{\text{clipp}}\right)\left[p\delta \left(\rho_{k}-L\right)+\left(1-p\right)\delta \left(\rho_{k}+L\right)\right] \end{array}$$

where *u*(*x*) is the unit-step function and *δ*(*x*) is the Dirac-delta function. Recall that *p* = *P*_D,SU_ or *p* = *P*_FA,SU_, depending on which case we are interested.

**Figure 3:**
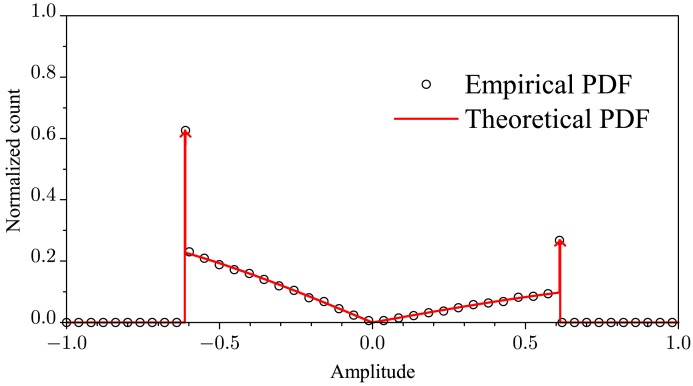
Empirical and theoretical pdfs of the noiseless received symbols transmitted from a single secondary user (SU).

To demonstrate the correctness of the Equation (17), Figure 3 shows the empirical (from the normalized histogram) and the theoretical pdf of the noiseless received symbols transmitted from an SU. We have used 500, 000 values of the corresponding random variable computed from Equation (13). The system parameters were arbitrarily chosen as *M* = 3, *K* = 1, *E*_b_ = 1, $C=2.9\sqrt{E_{\text{b}}}$ and *p* = 0.3. From this figure, the adherence between the empirical and the theoretical plot is clear. To avoid erroneous analysis, the continuous part of the pdf was estimated without the values corresponding to the discrete part, which are represented by impulses (arrows). The above value of *C* was computed according to the procedure described at the end of Section 2.2.

The pdf of $\rho ={\text{∑}}_{k=1}^{M}\rho_{k}$, which is associated with the histogram in Figure 1, will be the convolution of the *M* densities *f_ρk_*(*ρ_k_*) or the inverse Fourier transform of the product of the corresponding characteristic functions, under the assumption of independent channel gains and SU decisions. As far as we know, however, the derivation of such a pdf seems to be intractable, even numerically. Thus, below, we present the derivation of an approximate expression for such a pdf. The numerical results shown in the next section will unveil that this approximation is in fact very accurate.

Comparing Figure 1 with Figure 3, we see that the continuous part of the pdf in Figure 1 resembles a sum of weighted and shifted versions of the continuous part of the pdf in Figure 3. The discrete part of the pdf in Figure 1 is just given by the probabilities computed from Equation (8), weighted by 1 − *P*_out_. Then, we can approximate the pdf of *ρ* as:

(18) $$f_{\rho }\left(\rho \right)=\overset{M-1}{\underset{i=0}{\text{∑}}}\frac{P_{\text{out}}}{P_{\text{clipp}}}f_{\rho k}^{1}\left[\rho -\left(2i-M+1\right)L\right]w_{i}+\overset{M+1}{\underset{i=1}{\text{∑}}}P_{i}\left(1-P_{\text{out}}\right)\delta \left[\rho -\left(2i-M-2\right)L\right]$$

where:

(19) $$f_{\rho_{k}}^{1}\left(\rho_{k}\right)=\left[1-u\left(\left|\rho_{k}\right|-L\right)\right]\;\left[pg\left(\rho_{k}\right)+\left(1-p\right)g\left(-\rho_{k}\right)\right]$$

is the continuous part of *f_ρk_*(*ρ_k_*), and:

(20) $$w_{i}=\left(\begin{array}{c} M-1\\ i \end{array}\right)p^{i}{\left(1-p\right)}^{M-1-i}$$

and where, as before, *p* = *P*_D,SU_ or *p* = *P*_FA,SU_, depending on which case we are interested.

**Figure 4:**
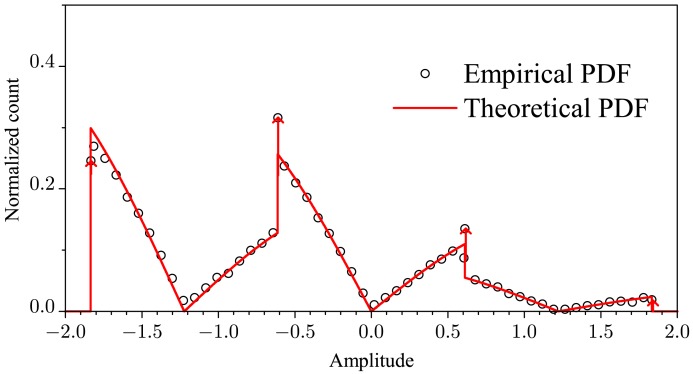
Empirical and theoretical pdfs of the noiseless received symbols from all SUs.

As the first evidence of the accuracy of Equation (18), Figure 4 shows the theoretical approximation of the pdf drawn from Equation (18) for *M* = 3, *K* = 1, *E_b_* = 1, $C=2.9\sqrt{E_{\text{b}}}$ and *p* = 0.3. A very close adherence is observed in this figure. More evidence of the accuracy of Equation (18) will be demonstrated by the numerical performance results in the next section. As in the case of Figure 3, the continuous part of the pdf shown in Figure 4 was estimated without the values corresponding to the discrete part and *vice-versa*.

From Figure 1 or from more detailed information given in [21], it is not difficult to see that under the decision based on the comparison of *r* in Equation (7) with the threshold defined in Equation (11), the probabilities of detection and false alarm at the FC, without clipping, can be computed from:

(21) $$P_{\text{D},\text{FC}}=\overset{M}{\underset{i=0}{\text{∑}}}Q\left[\frac{\lambda +\left(M-2i\right)L}{\sqrt{\frac{E_{\text{b}}}{2\Gamma_{\text{FC}}}}}\right]\times \left(\begin{array}{c} M\\ i \end{array}\right){P_{\text{D},\text{SU}}}^{i}{\left(1-P_{\text{D},\text{SU}}\right)}^{M-i}$$

(22) $$P_{\text{FA},\text{FC}}=\overset{M}{\underset{i=0}{\text{∑}}}Q\left[\frac{\lambda +\left(M-2i\right)L}{\sqrt{\frac{E_{\text{b}}}{2\Gamma_{\text{FC}}}}}\right]\times \left(\begin{array}{c} M\\ i \end{array}\right){P_{\text{FA},\text{SU}}}^{i}{\left(1-P_{\text{FA},\text{SU}}\right)}^{M-i}$$

where Γ_FC_ = *E*_b_/*N*_0_ is the average received SNR per bit at the fusion centre.

Taking into account the effect of fading and clipping, the probabilities of detection and false alarm can be computed from the average of Equations (21) and (22) over Equation (18), respectively leading to Equations (23) and (24). In these expressions, we have adequately moved the discrete part of *f_ρ_*(*ρ*) from the integrands to allow for numerical computations. The weights *w_i_* are calculated from Equation (20) for *p* = *P*_D,SU_ or *p* = *P*_FA,SU_ according to the desired probability, and $f_{\rho_{k}}^{1}\left(\rho_{k}\right)$ is defined in Equation (19).

(23) $$P_{\text{D},\text{FC}}=P_{\text{out}}\int_{-ML}^{ML}Q\left[\frac{\lambda -\rho }{\sqrt{\frac{E_{\text{b}}}{2\Gamma_{\text{FC}}}}}\right]\frac{1}{P_{\text{clipp}}}\overset{M-1}{\underset{i=0}{\text{∑}}}f_{\rho_{k}}^{1}\left[\rho -\left(2i-M+1\right)L\right]\;w_{i}d\rho +(1-P_{\text{out}})\overset{M}{\underset{i=0}{\text{∑}}}Q\left[\frac{\lambda +\left(M-2i\right)L}{\sqrt{\frac{E_{\text{b}}}{2\Gamma_{\text{FC}}}}}\right]\left(\begin{array}{c} M\\ i \end{array}\right){P_{\text{D},\text{SU}}}^{i}{\left(1-P_{\text{D},\text{SU}}\right)}^{M-i}$$

(24) $$P_{\text{FA},\text{FC}}=P_{\text{out}}\int_{-ML}^{ML}Q\left[\frac{\lambda -\rho }{\sqrt{\frac{E_{\text{b}}}{2\Gamma_{\text{FC}}}}}\right]\frac{1}{P_{\text{clipp}}}\overset{M-1}{\underset{i=0}{\text{∑}}}f_{\rho_{k}}^{1}\left[\rho -\left(2i-M+1\right)L\right]\;w_{i}d\rho +(1-P_{\text{out}})\overset{M}{\underset{i=0}{\text{∑}}}Q\left[\frac{\lambda +\left(M-2i\right)L}{\sqrt{\frac{E_{\text{b}}}{2\Gamma_{\text{FC}}}}}\right]\left(\begin{array}{c} M\\ i \end{array}\right){P_{\text{FA},\text{SU}}}^{i}{\left(1-P_{\text{FA},\text{SU}}\right)}^{M-i}$$

## 3. Numerical Results

Before presenting results regarding the comparison among the various fusion schemes, we first analyse the accuracy of the expressions derived in the previous section.

### 3.1. Accuracy of the Expressions for Computing the Probabilities of Detection and False Alarm

**Figure 5:**
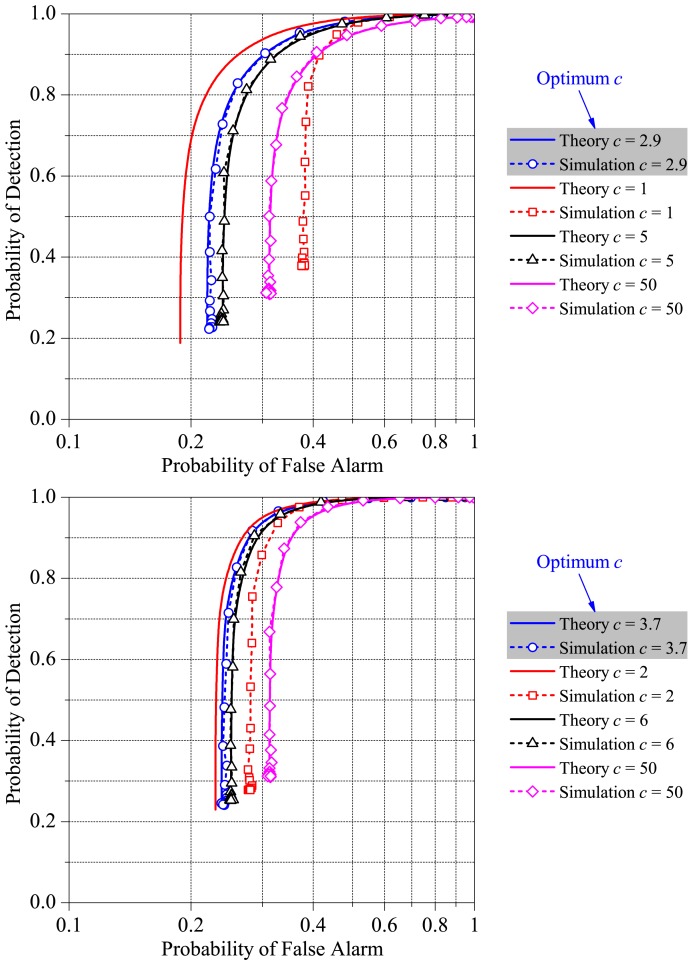
Theoretical and simulated ROC curves of the proposed fusion scheme for *M* = 3 (**Top**), *M* = 5 (**Bottom**), *K* = 1 and different clipping thresholds.

In order to allow for an analysis of the accuracy of Equations (21) and (22), Figure 5 shows theoretical and simulated ROC curves of our fusion scheme for *M* = 3, *M* = 5 and *K* = 1 and different clipping thresholds $C=c\sqrt{E_{\text{b}}}$. The received SNR at the SUs was arbitrarily set to Γ_SU_ = −5 dB and the average received SNR per bit at the FC was set to Γ_FC_ = 0 dB. First, we notice that a better accuracy of Equations (21) and (22) is achieved for large values of *c*, which is an obvious result, since large *c* means a small probability of clipping and, thus, small probabilities that the noiseless received symbols fall outside their awaited values, *i.e.*, small *P*_out_. However, large values of *c* lead to transmitted signals with high PAPR, which reduces the average Euclidean distance between the transmitted symbols, eventually reducing the detection performance at the FC. Recall that a high PAPR is the result of the attempt to compensate for low channel gains and that a resulting BPSK signal with high peaks will cause a compression on the original symbol position, so as to maintain the average energy *E*_b_; mathematically, high values of $C=c\sqrt{E_{\text{b}}}$ will lead to high values of *ξ* in Equations (21) and (22). On the other hand, very low clipping thresholds, while good from the perspective of the PAPR, also degrade performance. This is due the fact that a high *P*_out_ produces a high influence on the threshold-based decision rule proposed here, because a received symbol may cross the threshold even without noise. Finally, from Figure 5, it is also possible to observe that the procedure described at the end of Subsection 2.2 for determining *C* is indeed adequate: notice that with *c* = 2.9 for *M* = 3 and *c* = 3.7 for *M* = 5, *i.e.*, $C=2.9\sqrt{E_{\text{b}}}$ and $C=3.7\sqrt{E_{\text{b}}}$, respectively, the simulated performance is the best one among those shown, and the gap from the theoretical performance is small. This gap is even smaller in the case of c slightly above the optimum, and the performance is only slightly decreased; however, the PAPR is increased more noticeably, from around 3.2 for *c* = 2.9, to around 6.8 for *c* = 5 in the case of *M* = 3. For *M* = 5, the PAPR is increased from around 4.5 for *c* = 3.7, to around nine for *c* = 6. These values can be easily verified from the curve of *C versus* PAPR in Figure 2.

From above, we can conclude that the expressions, (21) and (22), are very accurate for predicting the spectrum sensing performance, for any clipping threshold value above the so-called optimum, which is computed according to the process described at the end of Section 2.2. Lower values for the clipping threshold make the pdf of *ρ* given in Equation (18) depart from the actual pdf. In other words, the approximated pdf in Equation (18) looses its accuracy for very low clipping threshold values, which are indeed useless from a practical standpoint due to the high resulting signal distortion and non-optimality of the overall spectrum sensing performance.

### 3.2. Performance Comparisons

In this section, we compare the performances of our simultaneous transmission reporting scheme with the scheme proposed in [20] and with the conventional reporting scheme in which the local decisions are sent via orthogonal channels. For this conventional scheme, if $P_{\text{D},\text{SU}}^{'}$ and $P_{\text{FA},\text{SU}}^{'}$ respectively denote the probability of detection and the probability of false alarm, taking into account the transmission errors for the local decisions, then:

(25) $${P^{\prime }}_{\text{D},\text{SU}}=P_{\text{D},\text{SU}}\left(1-P_{e}\right)+P_{e}\left(1-P_{\text{D},\text{SU}}\right)$$

(26) $${P^{\prime }}_{\text{FA},\text{SU}}=P_{\text{FA},\text{SU}}\left(1-P_{e}\right)+P_{e}\left(1-P_{\text{FA},\text{SU}}\right)$$

where *P*_e_ is the modulation-dependent and channel-dependent bit error probability. For BPSK modulation with coherent detection over the slow flat Rayleigh fading reporting channel with Ω = 1, this error probability is given by:

(27) $$P_{\text{e}}=\frac{1}{2}\left(1-\sqrt{\frac{\Gamma_{\text{FC}}}{1+\Gamma_{\text{FC}}}}\right)$$

The probabilities of detection and false alarm at the FC for the conventional scheme using the hard decision are, then,

(28) $$P_{\text{D},\text{FC}}=\overset{M}{\underset{l=K}{\text{∑}}}\left(\begin{array}{c} M\\ l \end{array}\right){{P^{\prime }}_{\text{D},\text{SU}}}^{l}{\left(1-{P^{\prime }}_{\text{D},\text{SU}}\right)}^{M-l}$$

(29) $$P_{\text{FA},\text{FC}}=\overset{M}{\underset{l=K}{\text{∑}}}\left(\begin{array}{c} M\\ l \end{array}\right){{P^{\prime }}_{\text{FA},\text{SU}}}^{l}{\left(1-{P^{\prime }}_{\text{FA},\text{SU}}\right)}^{M-l}$$

The system parameters adopted for the comparisons were *M* = 5 and *M* = 3 secondary users, for *K* = 1, *K* = [*M*/2] = 3 (resp. two) and *K* = *M* = 5 (resp. three) in the *K*-out-of-*M* rule. These values of *K* were chosen to configure the well-known decision fusion rules OR, majority-voting and AND, respectively. The received SNR at the SUs was arbitrarily set to Γ_SU_ = −5 dB, and the average received SNR per bit at the FC was set to Γ_FC_ = 0 dB and −5 dB. Each value of the channel gain *h_k_* was drawn from a zero-mean complex Gaussian distribution with an unitary second moment. The hypotheses *h*_0_ and *H*_1_ were considered equiprobable.

For convenience, hereafter, we identify the conventional scheme as the reference. When the original decision rule (2) is applied to the scheme proposed in [20], we refer to it as original MAP. When our new transmission and detection scheme is considered, we simply refer to it as new.

Each value on the receiver operating characteristic (ROC) curves shown in this section was obtained from 500,000 Monte Carlo events. Each event corresponds to sending a zero-mean white Gaussian distributed PU signal through *M* independent AWGN channels to the SUs and performing independent energy detections at the SUs from *N* = 100 received samples, for a given SU decision threshold. The individual SU's decisions were then sent to the FC through the reporting channel, where the final decision upon the sensed channel occupation was made depending on the analysed technique. The SU's decisions and the final decision at the FC were used separately for computing false alarm and detection rates, which are the estimates of the associated probabilities. Repeating the above procedure while varying the SU decision threshold traces-out the complete ROC curves.

**Figure 6:**
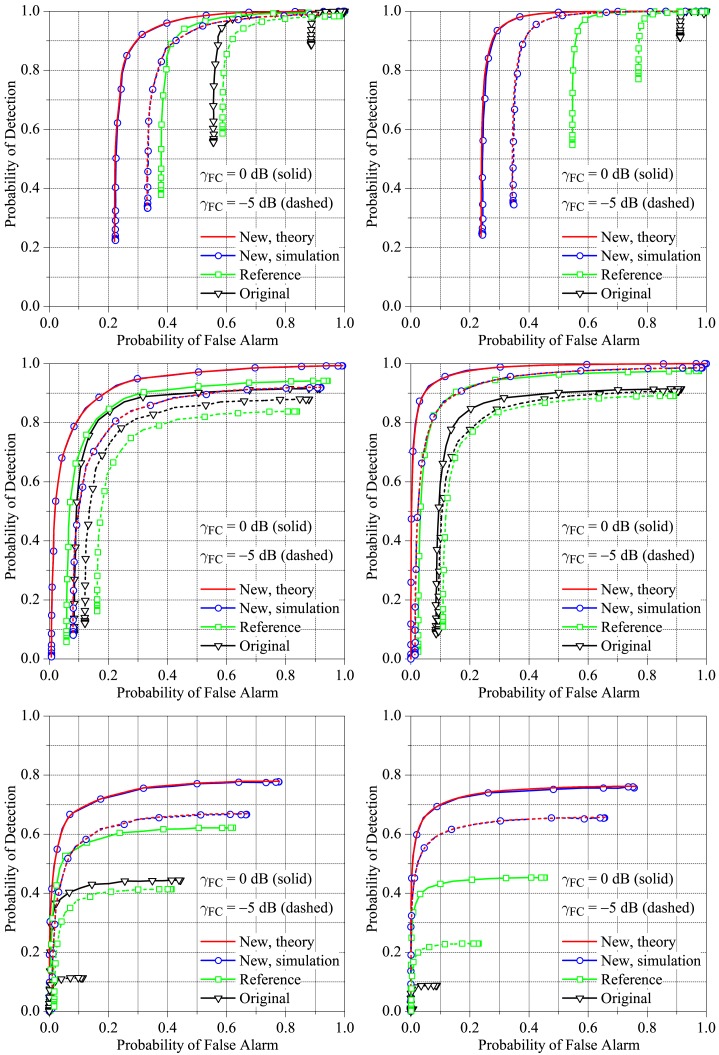
ROC curves at the fusion centre (FC) for *M* = 3 (**Left**), *M* = 5 (right), *K* = 1 (**Top**), *K* = [*M*/2] (**Middle**) and *K* = *M* (**Bottom**), *γ*_SU_ = −5 dB, *γ*_FC_ = 0 and −5 dB.

Figure 6, left, shows results for *M* = 3 and *K* = 1, 2 and 3, whereas Figure 6, right, shows results for *M* = 5 and *K* = 1, 3 and 5. In the case of *M* = 3, the clipping threshold is $C=2.9\sqrt{E_{\text{b}}}$. For *M* = 5, $C=3.7\sqrt{E_{\text{b}}}$. From these figures we first notice that our new fusion scheme outperforms the original and the reference schemes in all situations analysed. The gaps between the performances of our scheme and the original one can be very large, especially when the OR and AND rules are adopted. The performance gaps from our scheme and the reference one can also be very large, mainly when the OR and AND rules are adopted. Recall that the reference scheme is the one in which orthogonal transmissions from the SUs to the FC take place, occupying a larger amount of bandwidth or time resources as the number of SUs grow. We also notice from Figure 6 that the majority-voting (*K* = 2 and *K* = 3, respectively, for *M* = 3 and *M* = 5) results in the best performances of all fusion schemes. We also observe that the theoretical and simulation results are practically the same, attesting to the accuracy of the expressions derived throughout Section 2.

## 4. Conclusions

In this paper, we have proposed a new simultaneous transmission and decision scheme in which the task of channel estimation is shifted from the FC to the SUs, without the need for feeding back the estimates to the FC. The estimates are used at the SUs to pre-compensate for the reporting channel phase rotations and to partially compensate for the channel gains. This partial compensation is the result of signal clipping for PAPR control during the SU's reporting transmissions.

We have also suggested an approach for computing the optimum clipping threshold and showed that the resulting PAPR is not too high. Nevertheless, the PAPR can be easily reduced under the control of the clipping threshold, at the expense of a degradation in the performance of the spectrum sensing.

We have shown that our new fusion and decision scheme can produce large performance improvements over the original one proposed in [20], even outperforming the conventional reporting scheme in which orthogonal transmissions are used to report the SU's decisions to the FC. Our scheme also exhibits reduced implementation complexity when compared to the one proposed in [20], mainly due to a simpler decision rule and to a more feasible channel estimation process.

Expressions for approximating the probabilities of detection and false alarm were also provided and validated through simulations. These expressions produce very accurate results, as long as the clipping threshold value is equal to, or larger than, the optimal one. A method for computing this optimal threshold was also proposed and shown to be sufficiently precise.

Possible improvements of our technique can be envisaged if symbol probabilities and/or the sizes of the sets *D*_0_ and *D*_1_ are taken into account for computing the decision threshold. However, this would demand the knowledge or the estimation of the probabilities of detection and false alarm achieved by the SUs. Our threshold-based decision rule could also consider a possible imbalance between the probabilities of *H*_0_ and *H*_1_, which will produce a shift in the decision threshold, as well. To make use of this information, though, these probabilities should be known from the primary network or learned from past decisions in the secondary network.
